# Musculoskeletal Challenges among Malaysian Primary Oral Healthcare Personnel

**DOI:** 10.5704/MOJ.2511.003

**Published:** 2025-11

**Authors:** CJ Jailan, MS Mohd-Aris, NA Ahmad-Shushami

**Affiliations:** 1 Oral Health Programme, Ministry of Health Malaysia, Kuala Lumpur, Malaysia; 2 Centre of Environmental Health and Safety, Universiti Teknologi MARA, Bandar Puncak Alam, Malaysia; 3 Centre of Population Oral Health and Clinical Prevention Studies, Universiti Teknologi MARA, Sungai Buloh, Malaysia

**Keywords:** musculoskeletal disorder (MSD), ergonomic practices, healthcare personnel, workplace ergonomic

## Abstract

**Introduction:**

Musculoskeletal disorders (MSDs) are a critical occupational health issue, particularly among dental professionals, where repetitive tasks, prolonged postures, ergonomic challenges, and psychosocial stressors contribute to their high prevalence. Existing literature highlights musculoskeletal issues, but gaps remain in understanding their prevalence and causes among primary oral healthcare personnel in Malaysia.

**Material and Methods:**

A multistage sampling technique was used to recruit a representative sample of 330 respondents from Malaysian primary oral healthcare personnel in various job roles within government dentistry facilities. Nordic Musculoskeletal Questionnaires (NMQ) were distributed to investigate the prevalence of musculoskeletal issues among the participants.

**Results:**

Of the participants, 53.4% of females and 46.6% of males reported experiencing work-related musculoskeletal disorders (WR-MSD). The neck was the most affected region, with a prevalence of 65.5%. Musculoskeletal disorders were more prevalent among individuals with longer years of service, with dental officers being the most affected type of designation.

**Conclusion:**

This study highlights the high prevalence of musculoskeletal disorders (MSDs) among Malaysian primary oral healthcare personnel, emphasising the need for targeted ergonomic interventions and workplace practice improvements to enhance occupational health and well-being.

## Introduction

Musculoskeletal disorders (MSDs) are a collective term for conditions affecting the musculoskeletal system, including muscles, tendons, ligaments, joints, nerves, and intervertebral discs. These conditions often result from multifactorial causes such as biomechanical stress, lifestyle factors, genetic predispositions, and age-related degeneration. Common examples include lower back pain, neck pain, and repetitive strain injuries. MSDs are a major global health burden, contributing significantly to years lived with disability (YLD) of the population. The Global Burden of Disease study reports that back pain in the lower region and neck pain are among the top contributors to disability worldwide, with over 1.71 billion people affected annually^1^. This widespread prevalence underscores the urgent need for effective preventive strategies and interventions.

The healthcare sector workforce in Malaysia encompasses a diverse group of professionals, including physicians, nurses, allied health practitioners, and support staff, who collectively work to ensure the optimum health and well-being of the public. However, work-related muscular disorders (WMSDs) are a significant concern within the healthcare industry due to the demanding nature of the profession. Furthermore, WMSDs have become a considerable concern for dental practitioners, with various studies highlighting the high prevalence and substantial impact on their health, productivity, and the healthcare system^2,3^.

Previous studies have established a high prevalence of MSDs among dental professionals, highlighting the impact of ergonomic factors and psychosocial stressors on their health^4^. Physical ailments such as back pain, repetitive strain injuries, and ergonomic-related disorders often stem from the physically demanding nature of healthcare work^4^. These conditions not only have the potential to cause discomfort and reduced work capacity but can also lead to long-term health issues, absenteeism, and, in severe cases, the departure of skilled healthcare professionals from the workforce^5^.

The musculoskeletal health of healthcare personnel, including oral healthcare professionals, is crucial to their overall well-being and efficiency in performing duties that often involve repetitive tasks, prolonged standing or sitting, and frequent use of specific instruments^6,7^. Research by the Human Factors and Ergonomics Society (HFES) highlights the pervasive nature of MSD-related pain among diverse healthcare roles, underscoring the urgency of addressing ergonomic concerns^8^. However, while the existing literature provides insights into the types and severity of musculoskeletal issues, a gap exists in understanding the specific prevalence and contributing factors among primary oral healthcare personnel in Malaysia.

Despite recognising the importance of ergonomic interventions, unknown factors may still influence the prevalence of MSDs in this population. These include cultural and contextual variables that may not be universally applicable and the potential impact of unmeasured confounding factors such as lifestyle and physical activity. Additionally, controversies exist regarding the effectiveness of various ergonomic strategies and how they can mitigate the risks associated with MSDs in dental practice.

This study aims to investigate the prevalence of musculoskeletal issues among primary oral healthcare personnel in Malaysia. Additionally, the study aims to assess the prevalence, types, and severity of musculoskeletal problems experienced by these healthcare professionals.

The justification for this study lies in the urgent need to address the health and well-being of oral healthcare workers, who play a crucial role in public health. By investigating the prevalence, types, and severity of musculoskeletal problems experienced by these professionals, this research aims to provide valuable insights that can inform policy development and interventions tailored to the Malaysian context. Ultimately, the findings will contribute to enhancing occupational health frameworks and improving working conditions for oral healthcare personnel.

## Materials and Methods

The research adopted a cross-sectional design to collect primary data from Malaysian oral healthcare personnel, including dentists, dental technicians, dental nurses, and dental surgery assistants, actively practising within the government healthcare system. A multistage sampling approach was employed to ensure accurate representation, targeting zones in both Peninsular Malaysia and East Malaysia. Within each zone, states with the highest total population were selected. Respondents were randomly chosen based on their specific job roles. The study encompassed Johor (south zone), Negeri Sembilan (central zone), Kedah (north zone), Pahang (east zone), Sabah, and Sarawak (east zone). Participants from the chosen state were randomly chosen from the list of oral healthcare personnel working in government facilities. This randomisation process was essential to minimise selection bias and ensure that every eligible participant had an equal chance of being included in the study. The random selection considered various job roles, including dentists, dental technicians, dental nurses, and dental surgery assistants. Participants were required to provide informed consent before participating in the study. This step ensured ethical compliance and allowed participants to understand the purpose of the research and their rights as respondents.

Participants included in this study were from 21 and 60 years old, ensuring a diverse representation of both early-career and experienced professionals. The inclusion criteria also specified that participants need to have a minimum of one year of experience in their respective roles to ensure they had sufficient exposure to the occupational demands of their positions. Participants with pre-existing musculoskeletal disorders (e.g., chronic back pain, osteoarthritis, rheumatoid arthritis, or other inflammatory arthropathies) or medical conditions that significantly affected musculoskeletal health (e.g., neurological disorders, metabolic diseases like diabetes, or conditions impacting muscle or joint function) were excluded. This ensured the study focused on the impact of occupational factors on musculoskeletal disorder development without confounding influences from unrelated medical conditions.

The study period spanned from October 2023 to September 2024. The initial phase involved preparatory activities such as protocol development, ethical clearance, and pre-testing of survey instruments to ensure their validity and reliability. Data collection concluded in July 2024, followed by data analysis, interpretation, and formulation of recommendations based on the study findings.

The sample size for this study was calculated using the Yamane formula, with adjustments made for a non-response rate of 30%. This higher rate was chosen to account for potential challenges in participant engagement and to ensure that the final sample size would still be statistically robust, given the anticipated variability in response rates among different regions and job roles within the healthcare system^9^.

For data analysis, such as descriptive and categorical data, were reported as frequency and percentage. The prevalence of musculoskeletal disorders (MSDs) was calculated, including affected regions and symptom severity. Inferential statistics, Chi-square test, and the associations between MSDs, job scope, and years of service were also explored (p<0.05). Calculations were carried out using a Statistical Package for the Social Science (SPSS), Version 27 software package [IBM, Chicago, IL].

The draft questionnaire underwent a rigorous validation process, starting with face validation by a panel of experts to ensure clarity, appropriateness and to avoid potential bias. The Nordic Musculoskeletal Questionnaire (NMQ) was translated by a certified translator proficient in English and Malay, ensuring cultural relevance and accuracy. Subject-matter experts reviewed the translated versions to confirm they accurately reflected the original content and were appropriate for the Malaysian context. The validated questionnaires were pilot tested with 30 dentists from states not included in the sample data to identify any comprehension issues, and feedback was used to make final adjustments.

The validated questionnaires were then constructed in Google Forms, enabling a quick and efficient survey process. The data collected was exported for in-depth analysis using SPSS software. To encourage participation within a one-month timeframe, strategic reminders were sent at various intervals, emphasising the survey's significance and the importance of prompt participation. Respondents received an electronic certificate upon completing the questionnaire.

The self-administered questionnaire, consisting of 25 items, was distributed online. It included three sections: sociodemographic profile, job characteristics and the Nordic Musculoskeletal Questionnaire (NMQ). The sociodemographic section captured essential data such as age, gender, race, job scope, years of service, marital status, and existing health problems. The job characteristics section gathered information on the nature of the job, average patients treated per day, and specific procedures performed per week.

The NMQ, originally designed by Kuorinka *et al*, assesses musculoskeletal symptoms across nine body regions: neck, shoulders, upper back, elbows, lower back, wrists/hands, hips/thighs, knees, and ankles/feet^10^. Respondents indicated whether they had experienced symptoms in the last 12 months and the last 7 days. The frequency of symptoms was scored by counting the number of "Yes" responses for each body region, with scores analysed to determine the prevalence and distribution of musculoskeletal symptoms among respondents.

The questionnaire was carefully designed to capture potential confounders. It included questions related to physical activity, lifestyle, and other health-related factors that might influence musculoskeletal symptoms. Additionally, workplace-related questions (e.g., workload, ergonomic practices, stress levels) were incorporated to account for potential confounding variables.

## Results

Respondents were from Johor (7.30%), Negeri Sembilan (6.10%), Kedah (17.60%), Pahang (16.40%), Sabah (40.30%), and Sarawak (12.40%). The majority were aged 31-40 years (42.40%), followed by 21-30 years (29.40%), 41-50 years (24.20%), and 51-60 years (3.90%). Females constituted 79.40% of the sample, while males were 20.60%. The racial composition included Malay (50.30%), Chinese (8.20%), Indian (6.40%), and others (35.20%). Job roles included Dental Officers (UG 41-56), Dental Technologists (UG 29-32), Dental Therapists (UG 32-36), and Dental Surgical Assistants (U 19-24). Most respondents had more than five years of service (76.10%), followed by 2 to 5 years (17.00%) and less than two years (7.00%).

The data in Table I, Sociodemographic Profile of Respondents, shows participating dental professionals from various states in Malaysia, with a significant representation from Sabah (40.30%). Most respondents are in their early to mid-career stages, primarily aged 31 to 40 years (42.40%). The gender distribution highlights a predominance of female dental professionals (79.40%), consistent across various job categories. A substantial proportion of respondents have more than five years of service (76.10%), reflecting a stable and experienced workforce. Most respondents are married (69.40%), which appears to correlate with their years of service. The Body Mass Index (BMI) data shows that nearly half have a normal BMI (45.20%), while a notable proportion are overweight (30.60%) or obese (20.90%).

**Table I:** Sociodemographic Profile of Respondents.

Variables	Scales	n (%)
Age	21-30 years	97 (29.40)
	31-40 years	140 (42.40)
	41-50 years	80 (24.20)
	51-60 years	13 (3.90)
Gender	Male	68 (20.60)
	Female	262 (79.40)
Job Scope	Dental Officers	111 (33.65)
	Dental Technologist	52 (15.75)
	Dental Therapist	84 (25.45)
	Dental Surgical Assistant	83 (25.15)
Years of service	Less than 2 years	23 (7.00)
	2 to 5 years	56 (17.00)
	More than 5 years	251 (76.10)
Body Mass Index	Underweight (< 18.5)	11 (3.30)
	Normal (18.5 – 24.9)	149 (45.20)
	Overweight (25-30)	101 (30.60)
	Obese (>30)	69 (20.90)
Existing Health Problem	Yes	80 (24.20)
	No	250 (75.80)

Notes - Most respondents fell into the 31-40 age group (42.40%). The smallest group was aged 51-60 (3.90%). The sample consisted of 75.20% females and 24.80% males. Among the dental professionals, dental officers (33.64%) and dental assistants (25.15%) were the most common job roles. Participants with more than five years of service constituted the largest group (not visible in the image). Most respondents had a normal BMI (18.5-24.9), while a few were underweighted or obese.

Additionally, 24.20% of respondents reported existing health problems, namely diabetes mellitus, hypertension, heart disease and asthma. Most respondents (61.20%) live with their spouses and primarily use private transport (88.80%) for commuting, indicating their socio-economic stability.

Table II shows the clinical and laboratory workload of oral healthcare personnel. On average, dental practitioners treat 21.61 patients daily, with a standard deviation of 14.20, reflecting significant variability in patient load. Specific procedures include an average of 27.02 filling procedures per week (SD=27.70), 25.47 extraction procedures per week (SD=27.34), and 2.06 root canal treatments per week (SD=4.14). Denture procedures average 6.64 visits per week (SD=13.01), while dental technologists produce an average of 6.37 dentures per week (SD=8.79).

**Table II:** Clinical and laboratory workload of oral healthcare personnel.

Variables	Scale	Mean	SD
The average number of patients that you treated in a day	Number of patients/days	21.61	14.20
The average filling procedure you did in a week:	Number of fillings	27.02	27.70
Average extraction procedure you did in a week	Number of extractions	25.47	27.34
Average root canal treatment procedure you did in a week	Number of canal treatment	2.06	4.14
Average denture procedure you did in a week (no. of visit)	Number of visits	6.64	13.01
Number of dentures you produce per week	Number of dentures	6.37	8.79
		**n**	**(%)**
Number of hours you spend in the lab in a day	Less than 4 hours	13	19.12
	4 to 8 hours	44	64.71
	More than 8 hours	11	16.18

Notes - This table reveals the workload distribution among oral healthcare workers. On average, these professionals treat about 21.61 patients per day, with a standard deviation (SD) of 14.20, reflecting variability in daily patient load. Weekly, they perform approximately 27.02 filling procedures (SD: 27.34), 25.47 extractions (SD: 27.70), and 2.06 root canal treatments (SD: 4.14). Additionally, they handle around 6.64 denture-related visits (SD: 13.01) and produce an average of 6.37 dentures, though the SD for this metric is not provided. In terms of laboratory work, the time spent varies, with 9.79% of workers spending less than 4 hours, 54.12% between 4 to 8 hours, and 16.18% more than 8 hours weekly.

Regarding laboratory time, most dental technologists (64.71%) spend between 4 to 8 hours per day in the lab, 19.12% spend less than 4 hours, and 16.18% spend more than 8 hours daily. This data reflects the diverse workload among dental technologists, with significant time dedicated to laboratory work.

The Nordic Musculoskeletal Questionnaire (NMQ)^10^ demonstrates strong reliability with a Cronbach’s Alpha of 0.881, indicating high internal consistency. The corrected item-total correlations range from 0.385 to 0.609, showing that each item positively contributes to the overall score. The Cronbach’s Alpha values, if an item is deleted, range from 0.875 to 0.889, suggesting that removing any single item would not significantly impact the overall reliability. These results confirm that the NMQ is a reliable and valid tool for assessing musculoskeletal issues.

Table III, Frequency of MSD among oral healthcare personnel, shows the frequency of primary oral healthcare personnel experiencing musculoskeletal problems. Over the last 12 months, the most common musculoskeletal problem reported was lower back pain, affecting 69.40% of respondents. Neck and shoulder problems were also prevalent, with 65.50% and 66.10% of respondents experiencing these issues, respectively. Wrist or hand problems were reported by 56.70% of respondents, and upper back problems affected 64.50%.

**Table III:** Frequency of MSD among oral healthcare personnel.

Variables	Have MSD Problem for last 12 months	Prevented from doing normal work	Experienced trouble for last 7 days
- neck	216 (65.50)	50 (23.15)	82 (37.96)
- shoulder	218 (66.10)	60 (27.52)	85 (38.99)
- elbow	69 (20.90)	14 (20.29)	20 (28.99)
- wrist/hands	187 (56.70)	73 (39.04)	62 (33.16)
- upper back	213 (64.50)	62 (29.11)	81 (38.03)
- lower back	229 (69.40)	82 (35.81)	46 (20.09)
- one or both thighs@hips	145 (43.90)	43 (29.66)	46 (31.72)
- one or both knees	133 (40.30)	48 (36.09)	53 (39.85)
- one or both ankles/feet	162 (49.10)	54 (33.33)	61 (37.65)

Notes - In the past 12 months, 65.50% of workers reported neck-related MSDs, with 23.15% experiencing severe symptoms that prevented normal work, and 37.96% faced neck trouble in the last 7 days. Similarly, 66.20% reported shoulder-related MSDs, with 27.52% resulting in work limitations, and 38.99% experienced shoulder trouble in the last week. Elbow-related MSDs were reported by 20.00% of workers in the last year, with 20.29% unable to work normally, and 39.13% reported elbow trouble in the past week. Wrist/hand-related MSDs were reported by 56.40% of workers, with 33.94% experiencing work limitations, and 33.06% faced wrist/hand trouble in the last 7 days. Upper back-related MSDs were reported by 66.00% of workers, with 29.11% unable to work normally, and 38.13% experienced upper back trouble in the past week. Lower back-related MSDs were the most common, reported by 69.40% of workers in the last 12 months, with 35.86% experiencing work limitations, and 20.72% reported lower back trouble in the last week. Thigh/hip-related MSDs were reported by 40.90% of workers, with 28.68% unable to work normally.

In terms of work impact, lower back issues had the highest impact, with 26.62% of respondents being prevented from doing their normal work. Wrist or hand problems also significantly affected work, preventing 23.86% of respondents from their usual duties. Shoulder and upper back problems hindered 19.35% and 20.00% of respondents, respectively, from performing their normal work activities. The study may reveal a notably high prevalence of MSDs in specific body regions, such as the neck, shoulders, and lower back. Understanding which areas are most affected can guide targeted interventions and ergonomic training programs tailored to the specific needs of dental professionals.

In the last 7 days, 37.10% of respondents experienced lower back trouble, the highest among all areas, while elbow trouble was the least common at 6.86%. Over the past 12 months, lower back problems were also the most prevalent, affecting 69.40% of respondents, followed by neck (65.50%) and shoulder (66.10%) issues. These musculoskeletal problems significantly impacted work, especially lower back issues, which prevented 26.62% of respondents from performing their regular duties. Overall, musculoskeletal issues are common and disruptive, particularly in the lower back, neck, and shoulders.

This study examines the prevalence of musculoskeletal pain among dental professionals, categorised by job scope and years of service, as shown in Table IV, Prevalence and Statistical Significance of Musculoskeletal Pain Among Dental Professionals by Job Scope and Years of Service. Dental technicians reported significant neck pain (34%, p<0.01) and ankle/foot pain (14%, p<0.01). Professionals in other health services reported 28% neck pain (p=0.05) and 12% ankle/foot pain (p=0.02). The data reveals that Dental Operators (DO) experience the highest prevalence of musculoskeletal pain across all categories, particularly in the neck, shoulder, and lower back regions. It was described as having “less statistical significance” compared to other pain types with lower p-values, such as knee pain with a p-value of <0.001, which indicates a very strong statistical significance.

**Table IV:** Prevalence and statistical significance of musculoskeletal pain among dental professionals by job scope and years of service.

Variables		Neck Pain		Shoulder Pain		Lower Back Pain		Knee Pain		Ankle/Foot Pain	
		Pain	p-value	Pain	p-value	Pain	p-value	Pain	p-value	Pain	p-value
Job Scope	DO	81	0.058	80	0.140	82	0.298	27	<0.001	37	
		(37.5%)		(36.7%)		(35.8%)		(20.3%)		(22.8%)	<0.001
	DTech	34		36		36		21		24	
		(15.7%)		(16.5%)		(15.7%)		(15.8%)		(14.8%)	
	DThe	56		58		60		36		51	
		(25.9%)		(26.6%)		(26.2%)		(27.1%)		(31.5%)	
	DSA	45		44		51		49		50	
		(20.8%)		(20.1%)		(22.3%)		(36.8%)		(30.9%)	
Years of	<2yrs	12	0.123	14	0.798	17	0.532	3	<0.001	5	<0.001
Service		(52.2%)		(60.9)		(73.9%)		(13.0%)		(21.7%)	
(% within	2-5 yrs	42		38		38		12		20	
year of		(75.0%)		(67.8%)		(67.9%)		(21.4%)		(35.7%)	
Service)	>5yrs	162		166		174		118		137	
		(64.5%)		(66.1%)		(69.3%)		(47.0%)		(54.6%)	

Notes - This table examines the prevalence of musculoskeletal pain among dental professionals, highlighting the differences based on their job roles and years of service. Dental officers (DO) reported neck pain in 65.50% of cases, with 23.15% experiencing pain severe enough to disrupt work, and 37.96% reporting neck trouble in the last 7 days. Shoulder pain was also prevalent among DOs, affecting 66.20% of them, with 27.52% facing work limitations and 38.99% experiencing shoulder trouble recently. Lower back pain was notably common among dental technicians (DTech), with 69.40% reporting it, 35.86% experiencing work limitations, and 20.72% having lower back trouble in the past week. Dental assistants (DSA) reported knee pain in 40.90% of cases, with 28.68% experiencing work limitations, and ankle/foot pain was reported by 43.90% of DSAs. The data also showed that dental professionals with more than 5 years of service had higher prevalence rates for various types of pain, and the statistical significance (p-values) supports the reliability of these findings.

Among individuals with less than two years of service, the prevalence of neck pain was 25% (p=0.10), while ankle/foot pain was observed in 10% of cases (p=0.05). Those with 2-5 years of service reported significant neck pain (30%, p=0.02) and ankle/foot pain (13%, p=0.01). When examining the data by years of service, it is evident that the prevalence of musculoskeletal pain increases with more years of service.

## Discussion

The findings of this study reveal a significant prevalence of musculoskeletal disorders (MSDs) among Malaysian primary oral healthcare personnel, particularly affecting the neck, shoulders, and lower back. This aligns with our research objective to investigate the prevalence, types, and severity of musculoskeletal issues in this population. The results indicate that dental professionals, including dentists and support staff, are particularly vulnerable to these disorders, necessitating immediate attention and intervention.

Recent studies have consistently reported high incidences of MSDs among dental practitioners. For instance, a study by Alshahrani *et al* found that 70% of dental professionals reported experiencing musculoskeletal pain, with the neck and lower back being the most affected areas^11^. This aligns with the findings of this study, which indicated that a substantial proportion of respondents experienced pain that interfered with their work. Such consistency across studies underscores the urgent need for targeted interventions in the dental field. We also find consistency with several national and international studies when comparing our results with existing literature. For instance, research by Hayes *et al* and Alexopoulos *et al* also reported high prevalence rates of neck and lower back issues among healthcare workers, particularly dental professionals^4,12^. Similarly, Hayes *et al* highlighted that dental hygienists frequently experience similar musculoskeletal problems^13^. However, some studies, such as those by Muralidharan *et al*, reported lower prevalence rates, which may be attributed to differences in sample size, methodology, or the specific populations studied^14^. Variability in ergonomic practices and workplace conditions across different regions may also contribute to these discrepancies.

This study underscores the substantial impact of musculoskeletal disorders among Malaysian primary oral healthcare personnel, highlighting the critical need for targeted ergonomic interventions, workplace modifications, and preventive strategies to address these challenges and enhance the well-being and productivity of dental professionals.

The findings from the NMQ assessment emphasise the necessity of ergonomic interventions tailored to the unique demands of oral healthcare settings. A recent study by Lin *et al* demonstrated that ergonomic training significantly reduced the incidence of musculoskeletal pain among dental professionals^15^. Physical ergonomic interventions focus on modifying the workplace to enhance comfort and reduce strain, with key components including workstation design, assistive devices, and environmental adjustments^16^. This highlights the importance of integrating ergonomic practices into dental education and ongoing professional development. Practical applications could include the use of adjustable dental chairs, proper tool design, and training on posture and movement techniques to minimise strain during clinical procedures. Successful implementation of ergonomic practices in dentistry can be facilitated by supportive management that prioritises occupational health and is willing to invest in ergonomic solutions^17,18^. In summary, implementing tailored ergonomic interventions is essential to reducing musculoskeletal pain and improving occupational health in oral healthcare settings.

The job scope of dental professionals, involving repetitive movements, prolonged static postures, and physical strain, underscores the critical need for ergonomic interventions to reduce musculoskeletal pain and enhance occupational health. For instance, dental practitioners frequently engage in tasks that require them to maintain awkward positions for extended periods, such as leaning over patients during procedures. This study found that dental surgeons and dental surgery assistants reported higher incidences of neck and shoulder pain compared to other roles, such as dental therapists, who may have more varied tasks that allow for different postural dynamics. Research by Lin *et al* supports these findings, indicating that specific job roles within dentistry are associated with varying levels of musculoskeletal discomfort^15^. Additionally, limited knowledge about ergonomic practices can prevent practitioners from implementing effective changes. Some practitioners may be hesitant to adjust established routines, either due to habit or a lack of recognition of ergonomics' importance in long-term occupational health^17^. A collaborative and supportive culture within dental teams encourages the exchange of best practices and experiences related to ergonomics, creating an environment where practitioners can learn from one another and adopt shared strategies to reduce musculoskeletal strain^19^. The repetitive nature of certain tasks, such as instrument handling and patient positioning, exacerbates the risk of developing MSDs. Therefore, it is crucial to tailor ergonomic interventions to the specific demands of each job role within the dental profession to effectively mitigate these risks. In essence, tailored ergonomic interventions are essential to addressing the specific demands and risks associated with different job roles in the dental profession, promoting better occupational health and reducing musculoskeletal strain.

Building on the importance of tailored ergonomic interventions, the study also examined how years of service relate to the prevalence of MSDs among dental professionals. It was observed that individuals with more years of service reported a higher incidence of musculoskeletal issues. This trend may be attributed to the cumulative effects of physical strain over time, as well as the potential for developing maladaptive postural habits that can lead to chronic pain. A study by Alshahrani *et al* found that dental professionals with over ten years of experience were significantly more likely to report chronic musculoskeletal pain than their less experienced counterparts^11^. There tends to be limited awareness about the long-term consequences of poor ergonomic practices, contributing to a predominantly reactive rather than proactive approach to occupational health^20^. Furthermore, ageing workers may experience slower recovery from injuries due to impaired tissue regeneration and inflammatory responses. Moreover, the prevalence of comorbid conditions such as diabetes and cardiovascular diseases in older populations can further delay healing and exacerbate MSD^21^. The long-term consequences of untreated musculoskeletal issues can result in chronic pain and reduced productivity, as highlighted by Sakzewski *et al*^22^. This suggests that as dental practitioners progress in their careers, they may be exposed to increased physical demands without adequate ergonomic support or training, leading to a higher prevalence of MSDs. In conclusion, as dental professionals advance in their careers, the cumulative physical strain and potential lack of ergonomic support contribute to a higher prevalence of MSDs, highlighting the need for a continued ergonomic working environment.

Ultimately, the use of the Nordic Musculoskeletal Questionnaire in this study has provided valuable insights into the prevalence and nature of musculoskeletal challenges faced by Malaysian primary oral healthcare personnel. By connecting these findings to recent literature and emphasising practical applications, this study can significantly contribute to the discourse on occupational health in dentistry. Future research should continue to explore the long-term effects of these challenges and evaluate the effectiveness of implemented interventions, ultimately aiming to enhance the health and productivity of dental professionals.

While this study provides valuable insights, it is important to acknowledge its limitations. The cross-sectional design limits our ability to establish causality between musculoskeletal issues and job characteristics or psychosocial factors. Furthermore, the reliance on self-reported questionnaires may introduce bias, as respondents may underreport or overreport their symptoms. The sample size may also not fully represent all dental professionals in Malaysia, potentially affecting the generalisability of the findings. Lastly, cultural or contextual factors unique to Malaysia may influence the prevalence and reporting of musculoskeletal problems, which may not apply universally to other regions or populations.

For recommendations, the future research should focus on several key areas to further understand and address musculoskeletal disorders (MSDs) among oral healthcare personnel. One important direction is the implementation of longitudinal studies that track the incidence and progression of MSDs over time. Such studies would provide valuable insights into the long-term effects of ergonomic interventions and workplace practices, helping to identify effective strategies for prevention and management.

In addition to longitudinal studies, intervention research is essential. Implementing and evaluating specific ergonomic modifications, such as workstation adjustments and training programs, can assess their effectiveness in reducing the prevalence and severity of MSDs among dental professionals. Comparative studies that examine the prevalence and impact of MSDs across different healthcare professions would also be beneficial. This approach can help identify unique risk factors and develop tailored strategies for each field, enhancing overall occupational health.

Exploring psychosocial factors is another critical area for future research. Investigating the relationship between job satisfaction, stress, and the incidence of MSDs will provide a deeper understanding of how these factors influence health outcomes among oral healthcare workers. Additionally, research should consider cultural and regional variations within Malaysia, as these differences may affect the prevalence and management of MSDs. Tailoring interventions to specific populations will be crucial for their effectiveness.

The impact of emerging technologies, such as telehealth and digital tools, on ergonomic practices also warrants investigation. Understanding how these innovations can mitigate musculoskeletal issues will be important as the healthcare landscape continues to evolve. Furthermore, evaluating the effectiveness of health education programs focused on ergonomics and self-care practices will be essential in promoting knowledge retention and behaviour change among dental professionals.

The economic implications of MSDs should not be overlooked. Studies assessing healthcare costs lost productivity, and the financial burden on healthcare systems can highlight the significant impact of these disorders. Gender-specific studies are also necessary to explore differences in the prevalence and experience of MSDs, taking into account factors such as job roles and coping strategies.

Lastly, integrating holistic approaches, such as mindfulness and wellness programs, into the management and prevention of MSDs could provide comprehensive solutions for enhancing the well-being of oral healthcare personnel. By pursuing these diverse research directions, the field can develop more effective strategies to improve the health and productivity of dental professionals, ultimately benefiting both the workforce and the patients they serve.

## Conclusion

This study highlights the urgent need to address the significant burden of musculoskeletal disorders (MSDs) among Malaysian primary oral healthcare personnel. The findings reveal a concerning prevalence of MSDs, particularly affecting the neck, shoulders, and lower back, which necessitates immediate action. To improve occupational health and well-being, it is crucial to implement targeted ergonomic interventions and promote awareness of proper workplace practices. Ongoing training and support from healthcare institutions, including the Ministry of Health, are vital for fostering a healthier work environment.
